# Biomimetic Structure and Surface for Grasping Tasks

**DOI:** 10.3390/biomimetics9030144

**Published:** 2024-02-27

**Authors:** Jingyang Li, Fujie Yin, Yu Tian

**Affiliations:** 1 State Key Laboratory of Tribology, Tsinghua University, Beijing 100084, China; 2 Xingjian College, Tsinghua University, Beijing 100084, China

**Keywords:** biomimetic structure and surface, grasping tasks, gripper and robot

## Abstract

Under water, on land, or in the air, creatures use a variety of grasping methods to hunt, avoid predators, or carry food. Numerous studies have been conducted to construct a bionic surface for grasping tasks. This paper reviews the typical biomimetic structures and surfaces (wedge-shaped surface, suction cup surface and thorn claw surface) for grasping scenarios. Initially, progress in gecko-inspired wedge-shaped adhesive surfaces is reviewed, encompassing the underlying mechanisms that involve tuning the contact area and peeling behavior. The applications of grippers utilizing this adhesive technology are also discussed. Subsequently, the suction force mechanisms and applications of surfaces inspired by octopus and remora suction cups are outlined. Moreover, this paper introduces the applications of robots incorporating the principles of beetle-inspired and bird-inspired thorn claw structures. Lastly, inspired by remoras’ adhesive discs, a composite biomimetic adhesive surface is proposed. It integrates features from wedge-shaped, suction cup, and claw thorn surfaces, potentially surpassing the adaptability of basic bioinspired surfaces. This surface construction method offers a potential avenue to enhance adhesion capabilities with superior adaptability to surface roughness and curvature.

## 1. Introduction

In movies, Spider-Man can crawl vertically on walls with different roughness, such as glass and brick, or even cling upside down. In reality, organisms like geckos, beetles, and octopuses also exhibit remarkable performance on surfaces with varying roughness and curvature, achieving effective adhesion in both normal and tangential directions. This enables them to swiftly move during hunting or when evading predators, as well as to rest on surfaces for extended periods. These exceptional abilities arise from the diverse structures evolved in biology, enabling outstanding interfacial interaction forces, including friction, van der Waals forces, mechanical interlocking forces, and vacuum adhesion forces. However, due to manufacturing limitations, bioinspired adhesive surfaces and structures fall short of replicating the exact performance of biological surfaces. Their strength is significantly lower, primarily providing unidirectional bearing, and their adaptability to rough surfaces and curvature is often constrained by the featured length in manufacturing. Therefore, achieving more biological surface functionalities, such as omnidirectional bearing and higher adaptability to roughness, becomes crucial, given the current manufacturing capabilities.

**Figure 1:**
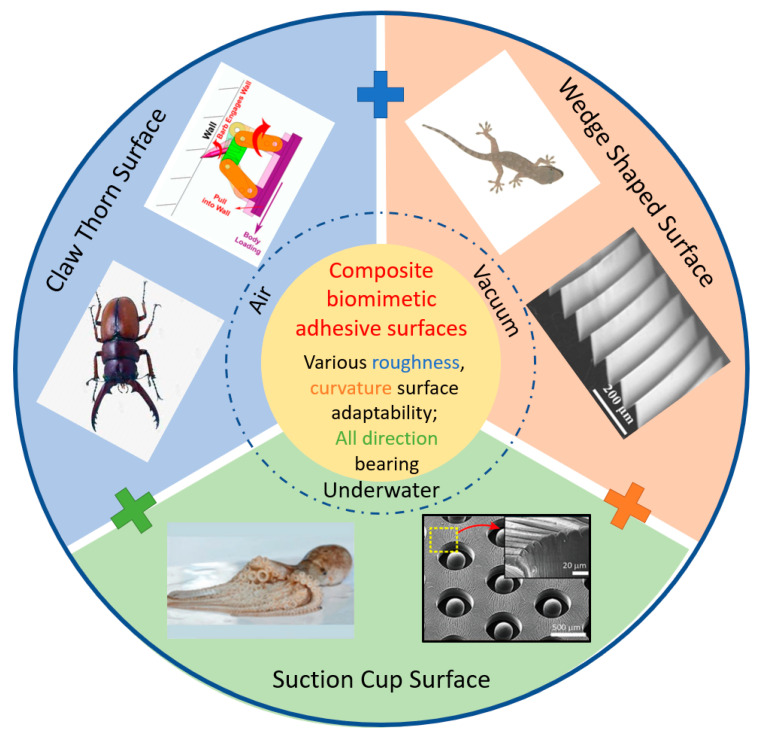
Biomimetic structure and surface for grasping. The wedge-shaped surface, suction cup surface and claw thorn surface find extensive applications in grasping tasks performed underwater, in vacuum environments, and in the air. With the combination of the bioinspired surface, composite biomimetic adhesive surface is possible to be attached to various roughness and curvature target surfaces with all-direction bearing. Adapted with permission from [8]. Copyright 2005, Springer-Verlag Berlin Heidelberg. Adapted with permission from [9]. Copyright 2015, WILEY-VCH Verlag GmbH & Co. Adapted with permission from [10]. Copyright 2021, IEEE. Adapted with permission from [11]. Copyright 2019, American Chemical Society.

Bioinspired structures and surfaces have certain limitations in terms of the usage environments, surface roughness, and curvature. They are typically designed for specific adhesion environments, with pressure adhesion often used in atmospheric or underwater high-pressure conditions [1,2], claw-like attachment structures suitable for air environments, and gecko-inspired adhesive surfaces often used in clean environments such as air or vacuum [3] (Figure 1). Additionally, the adaptability of bioinspired structures and surfaces to surface roughness varies. Pressure adhesion surfaces require relatively small surface roughness compared to the size of the suction cup; otherwise, it is challenging to generate a seal and create internal negative pressure [4,5]. Claw-like attachment structures are suitable for relatively rough surfaces, making mechanical interlocking between the claws and the target surface more achievable [6]. Gecko-inspired adhesive surfaces are suitable for relatively smooth surfaces, and excessive surface roughness leads to reduced contact quality, thereby diminishing the strength of the interfacial forces [7].

In-depth research on bio-switchable adhesion phenomena will accelerate the development of artificial surfaces with ideal adhesive functionalities. Pioneering studies have demonstrated many biomimetic array surfaces with excellent interfacial adhesion performance, including wedge-shaped arrays based on van der Waals forces, claw-like arrays benefiting from mechanical interlocking forces, and suction cup arrays relying on pressure differences. To further enhance the adhesion performance and adaptability, designing appropriate array structures has become a crucial strategy for practical applications. For example, inclined arrays, layered arrays, and intelligent adhesives based on smart materials exhibit switchable adhesion and the ability to adapt to rough surfaces to some extent. It is noteworthy that potential applications have been reported, including wearable electronic devices [12], integrated circuit manufacturing [13,14,15], and adhesion in microgravity environments [3,16].

Recent developments in biomimetic structures and surfaces for grasping tasks are comprehensively reviewed in this paper. The discussion begins with an exploration of the progress in gecko-inspired wedge-shaped adhesive surfaces, elucidating the underlying mechanisms that govern the tuning of the contact area and peeling behavior. Subsequently, the applications of grippers utilizing this technology are highlighted. This paper also provides a summary of the suction force mechanism and applications pertaining to octopus-inspired and remora-inspired suction cup surfaces. Moreover, the applications of robots and the principles underlying beetle-inspired and bird-inspired thorn claw structures are introduced. Finally, inspired by the adhesive discs of remoras, a composite biomimetic adhesive surface is introduced to enhance adhesion capabilities. By incorporating elements from the basic bioinspired surface (wedge-shaped surface, suction cup surface, and claw thorn surface), the composite biomimetic adhesive surface may demonstrate superior adaptability to surface roughness and curvature radius, as well as the ability for multidirectional bearing—attributes probably not present in the basic bioinspired surface.

## 2. Wedge-Shaped Adhesive Surface

The strong adhesion ability of the gecko foot allows it to steadily and rapidly crawl on a variety of complex surfaces. The feet of geckos have a rich multilevel structure. Each gecko toe has about 20 rows of lamellas, and each lamella possesses about 20 setal arrays. A single seta is about 110 μm long, with a diameter of approximately 4–6 μm. The seta is divided into 100–1000 spatula-shaped structures, each with a length of about 300 nm and a thickness of 5 nm [17,18]. The van der Waals forces between these spatulas and the substrate, capillary forces and chemical bonds are thought to be the main origin of adhesion [19,20,21]. Related studies have shown that gecko foot adhesion has the following characteristics: (a) anisotropy, (b) strong adhesion and low detachment force, (c) can be reused on a variety of substrate surfaces, (d) rate-dependent, (e) self-cleaning, and (f) self-adhesive resistance [4]. These characteristics show a wide range of applications in gripper design, climbing robots, space debris capture, etc., and thus, they have inspired the design of a micro-patterned dry adhesive surface.

The basic idea of a micro-patterned dry adhesive surface is to form arrays of fibers with a certain shape on a flat, soft surface. The adhesion force is generated by the contact between the fibers and the substrate. The fiber shape greatly affects the surface properties. Among many shapes, wedge-shaped fibers have a particularly good performance in controllable adhesion. Applying tangential forces to a surface with a wedge-shaped structure increases the contact area between the fiber and the substrate, thereby increasing the adhesion strength. When the tangential force is removed, the contact area is reduced and the adhesion strength decreases, which causes low detachment force. This makes wedge-shaped adhesive surfaces widely used in gripper design, climbing robots, space debris capture and many other fields.

### 2.1. Mechanism

To further clarify the adhesion mechanisms between the gecko toe and the substrate, it is vital to establish the corresponding adhesion models. Because the spatulae of gecko feet are essentially thin and flat, research on the adhesive properties of gecko feet is closely related to adhesive tapes [22], such as the Kendall model and peel zone model.

#### 2.1.1. Origin

Researchers have proposed several hypotheses to explain the origin of gecko adhesion, such as electrostatic interaction, vacuum, etc. Autumn et al. measured the force generated by a single foot hair, providing direct evidence of gecko adhesion based on the van der Waals forces [20]. It has also been widely recognized that in a humid environment, the sole of the gecko foot will also produce surface tension adhesion with the liquid on the surface of the substrate, and its effect on the total adhesion strength is equivalent to the magnitude of the van der Waals force [19,23]. Recently, Singla et al. proved that a chemical bond was formed between the soles of a gecko’s feet and the sapphire substrate through spectral analysis, and they speculated that the dominant force between the soles of the gecko’s feet and some surfaces was acid–base action, which was related to some oils secreted by the soles of the gecko’s feet [21].

#### 2.1.2. Kendall Peel Model

**Figure 2:**
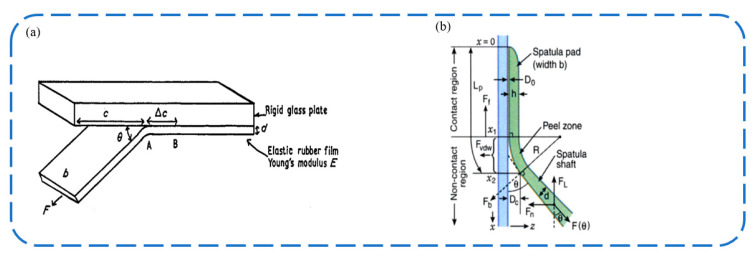
Models for a wedge-shaped adhesive surface. (**a**) Schematic diagram of Kendall model [24]. Adapted with permission from Ref. [24]. 1975, IOP Publishing Ltd. (**b**) Tian et al. divided adhesive stripping into three regions: contact zone, peel zone, and non-contact zone [26]. Adapted with permission from Ref. [26]. Copyright 2006, the National Academy of Sciences of the USA.

The Kendall peel model considers the debonding process as the problem of an elastic film stripping from a rigid substrate at a certain angle. As shown in Figure 2a, an elastic film with a thickness of d and a width of b is bonded to the rigid substrate. The external force F pulls the elastic film along the θ angle, causing the bonding zone length to decrease by Δc. The energy change in this process is divided into three parts: the elastic potential energy $\frac{F^{2}\Delta c}{2bdE}$; the change in adhesion energy caused by the reduction of the bonding area −bRΔc, where R represents the adhesion energy per unit area, which can be determined by experiment; and the work performed by the external force $F\Delta c\left(1-\cos\theta \right)$. Then, we have [24]:

$$-bR\Delta c+F\Delta c\left(1-\cos\theta \right)+\frac{F^{2}\Delta c}{2bdE}=0$$

This theory provides the relationship between the peel force F and the peel angle θ from a macroscopic perspective, and it explains the anisotropy and low detachment force of gecko feet to a certain extent. However, this model lacks physical images at the microscopic level. In addition, the elastic thin layer hypothesis is also oversimplified, and the actual gecko bristles have more complex shapes and hierarchical structures.

#### 2.1.3. Peel Zone Model

As shown in Figure 2b, the peel zone model is a model proposed by Tian et al. [25,26] to describe the peel behavior of tape at a peel angle of less than 90 degrees. Assuming that the tape is stripped at an angle of θ, the elastic energy term caused by the stress deformation of the tape is ignored, and only the surface energy is considered. Regardless of the relationship between the normal adhesion strength and the normal displacement in the peel zone, it is considered that the normal adhesion strength is a constant value. Then, the normal force $F_{n}$ is proportional to the area of the peel zone S=Rθb, where b is the tape width. The shape of the tape in the peel zone is approximated as a curvature circle, and the normal distance at the boundary of the peel zone is assumed to be a constant value. When the normal distance exceeds this value, there will be no interaction between the tape and the substrate. So we have: $R\propto \frac{1}{1-\cos\theta }$. Considering the overall balance of the tape, $F_{n}=F\sin\theta$. By integrating the above formula, we have: $F\propto \frac{\theta }{\sin\theta \left(1-\cos\theta \right)}$. The peel zone model is more consistent with the results of the tape peeling test than the Kendall model. Pesika et al. used the peel zone model to analyze the detachment process of geckos and proposed a mechanism whereby geckos achieve low detachment force by changing the peel angle and increasing the peel zone area through the tangential motion of setae [25,26].

In addition to the above models, there are many other studies on the mechanism of stripping. Based on the peel zone model, Zhou et al. proposed an extended peel zone model considering the influence of peel velocity [27]. Li et al. divided a tape into the adhesive layer and backing layer, and they simulated it with spring, respectively. Through force analysis, the differential equation was listed, the exact shape of the peel zone was calculated, and the Kendall model was explained from force analysis [28]. He et al. considered the influence of the hyperelastic effect and bending effect, and they used the variational method to offer the analytical formula of the peeling force in the stable peeling process, which can satisfactorily simulate the process of the peeling of gecko [22,29]. However, the above studies are about the peeling of thin films. More modeling work is needed to include structural features at higher hierarchical levels, and ultimately, the entire gecko [22].

### 2.2. Surface Capability

#### 2.2.1. Basic Capability

So far, the methods for manufacturing wedge-shaped dry adhesive surfaces can be roughly divided into three types [30,31,32]. (1). Etching and casting. The polymer is cast into an etched mold and then released. This method usually includes a series of complex procedures, including substrate preparation, spin-coating, baking, exposure, development, etc., for the cleanliness of the operating environment is required. (2). Vapor phase growth. Chemical vapor deposition is used to make nanotubes or nanowires. Manufactured surfaces are usually between millimeters and centimeters in size, and surfaces of larger sizes can only be achieved by a combination of small pieces. (3). Ultra-precision diamond cutting. The wedge-shaped mold is made by diamond cutting, and the wedge-shaped adhesive surface is obtained by polydimethylsiloxane. At present, most wedges have a surface scale of 50–100 um, and the wedge angle is between 30–50 degrees [30,31,32]. The characterization of the surface properties is usually achieved through three experiments: load–drag–pull; load–pull; and longevity test to examine its direction and repeatability [31]. As an example of the excellent properties of the wedge surface, the wedge surface prepared by Parness et al. [31] can be reused more than 30,000 times, and the shear strength reaches 17 kPa in the main direction and decreases to 10 kPa in the opposite direction.

#### 2.2.2. Shape Optimization

Optimization design can be roughly divided into two main aspects. The first is the mushroom shape optimization, where the fiber structure has rotation symmetry and the optimization goal is to maximize the adhesion [33,34,35,36]. The second is wedge shape optimization, where the fiber structure is asymmetric and the optimization goal needs to comprehensively consider controllable adhesion (that is, strong adhesion and low detachment force) and the adhesion strength [37]. There is relatively little research on wedge structure optimization. The reason may be that the selection of shape parameters is more complicated and difficult because the structure no longer has rotational symmetry. Moreover, the advantages of the wedge-shaped surface are anisotropy and controllable adhesion, so the optimization goal is better controllability. However, it is difficult to quantify the degree of anisotropy and controllability, which poses a challenge to wedge-shaped adhesive surface optimization.

Many asymmetrical shape designs have appeared. In addition to the most classic wedge structure, there are ladder structures made by Gwon et al., and on this basis, the addition of smooth arc layers to the boundary of contact with the interface [38]; the triangular flat bottom structure adopted by Kwak et al. [39]; improved on the basis of the mushroom type, so that the left and right radii are an inconsistent two-radius structure [40]. And inspired by the gecko hierarchy design of the triangular prism + end rectangular block structure [41], etc. The above studies are all about some regular and specific geometric shapes, which are easy to fall into local optimal solutions. In fact, the optimal shape boundary is not necessarily a simple combination of straight lines and arcs but may be a more general curve. Yongtae et al. applied deep learning and genetic optimization methods to anisotropy optimization [37]. Optimized fiber strength under different neck constraints was produced using 3D printing, with an improvement in adhesion strength between 48% and 169%.

### 2.3. Applications

To design a flexible, robust and universal gripper is always the goal. The traditional rigid gripper increases the friction force by increasing the pressure, so as to grasp, move and release. However, on the one hand, the robot’s grasp is only suitable for objects of its own scale, and it is powerless for flat or slightly curved surfaces. On the other hand, excessive pressure on fragile items can easily cause surface damage. The gripper based on the dry adhesive surface can solve the above problems well. Dry adhesives are generally soft materials, and greater adhesion can be achieved by applying a small normal preload. However, the strong adhesion of traditional dry adhesives also makes the release process difficult and reduces the controllability of the adhesion grabbing process. Wedge-shaped adhesive surfaces, as mentioned above, have excellent properties of “shear-controlled adhesion” and are therefore widely used in gripper design.

**Figure 3:**
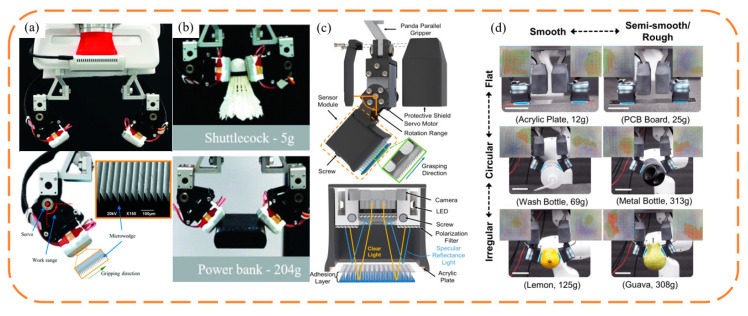
Schematic structure and photo of the clamping of objects of different shapes by Viko [10] and Viko2.0 [42]. adapted with permission from Ref. [10]. Copyright 2021, IEEE. Ref. [42]. Copyright 2018, IEEE. (**a**) Viko applies a wedge-shaped adhesive layer to the surface of the parallel gripper. (**b**) Clamping of objects of different shapes and weights by Viko. (**c**) Schematic structure of Viko2.0. (**d**) Viko2.0 clamping objects of different shapes and weights.

A common wedge-shaped surface-inspired gripper is to attach a wedge-shaped adhesive surface to the surface of the original manipulator and change the contact area between the adhesive layer and the object by controlling the tangential movement of the adhesive layer, so as to achieve controllable adhesion. In order to achieve stable control of the grasping process, the evaluation of the contact area during the grasping process is crucial, so it is necessary to configure the haptic sensors. The non-stretchability of conventional capacitive sensors hinders the flexibility of the adhesive layer. Viko, a gripper developed by Chosen Peng et al. [10], combines the wedge surface with a parallel gripper (Figure 3a,b), adopts a vision-based tactile sensor, and obtains information about the contact area during the adhesion process from visual information through a dense inverse search (DIS) algorithm. Given a series of shear forces, the corresponding area data are obtained, and the empirical relationship between the shear forces and area is obtained by fitting them together. In the clamping process, the sensor realizes the evaluation of the contact area and shear force, so as to realize the feedback control of the motor, and makes timely adjustments to the grasping angle and preload of the gripper according to the grasping process, improving the stability and versatility of the gripper. Viko enables adaptive grasping of a range of everyday objects. On this basis, Viko2.0 was developed, replacing the wedge surface with a layered adhesive composed of columns and wedges, which improved the normal adhesion by 1.5 times (Figure 3c,d). Added slip detection during initial contact prevents failure caused by slip during grasp [42].

**Figure 4:**
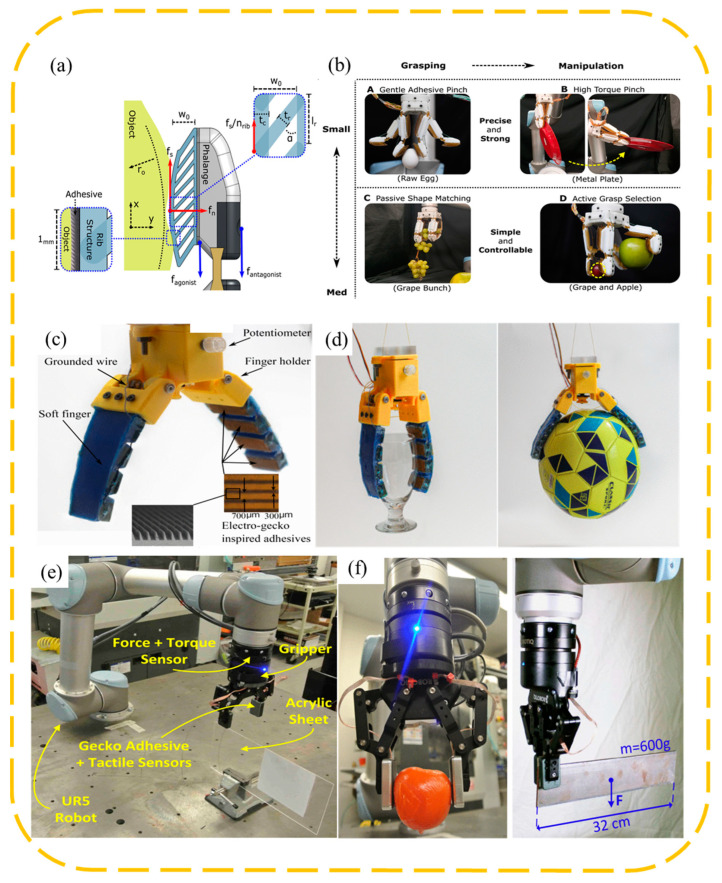
(**a**,**b**) Attach a wedge-shaped adhesive layer to a soft mechanical hand [43]. Adapted with permission from Ref. [43]. Copyright 2021, Ruotolo et al. (**c**,**d**) Attach a wedge-shaped adhesive layer to a multi-finger mechanical hand. Achieve flexible grasp of a variety of shapes [44]. Adapted with permission from Ref. [44]. Copyright 2020, IEEE. (**e**,**f**) The surface of the parallel manipulator is provided with a wedge-shaped adhesive layer, grasping a variety of objects [45]. Adapted with permission from Ref. [45]. Copyright 2018, IEEE.

FarmHand, developed by Wilson et al. [43], applies the wedge-shaped adhesive surface to the multi-finger gripper (Figure 4a,b). By designing the shape of the elastic rib and controlling the movement of the knuckles, it realizes the simultaneous grasping of a single grape and apple, which greatly improves the versatility of the manipulator. Alizadehyazdi et al. [44] combined the electrostatic adhesive with the wedge-shaped adhesive surface and applied it to the soft gripper, significantly improving the bonding strength and adaptability to surfaces of different curvatures (Figure 4c,d). Roberge et al. [45] pointed out that if the gripper needs to apply shear in only one direction, it is very effective to tilt the wedge surface in the same direction as the force applied (Figure 4e,f). However, due to the complex and diverse shapes of the object to be grabbed, the adhesive layer on the surface of the gripper needs to resist forces and torques in multiple directions, so it is best not to have a single adhesive orientation. The “V” configuration strikes a good balance between performance and ease of manufacture.

**Figure 5:**
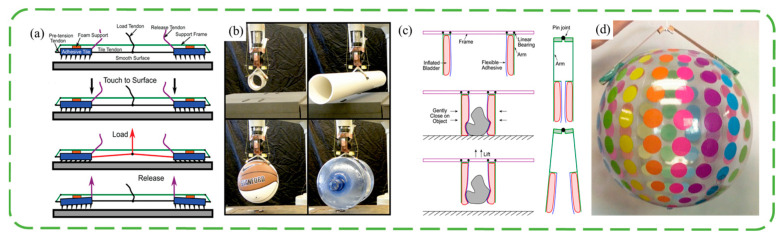
Schematic structure and photo of grasping objects by a double-tile gripper [47] (**a**,**b**) and soft air bag gripper (**c**,**d**) [50]. Adapted with permission from Ref. [47]. Copyright 2015 Hawkes et al. Adapted with permission from Ref. [50]. Copyright 2015, IEEE.

In addition to the additional adhesive layer on the surface of the original manipulator, there are many grippers designed directly from the wedge-shaped adhesive surface. This type of gripper is usually composed of a sticky tile and an actuator, and often, the target is dynamically attached. Two-tile designs are more common. The surfaces of the two tiles are pasted with wedge-shaped adhesive layers in opposite directions, and the tiles are connected with tendons. The angle of the loading tendon controls the ratio of normal force to shear force, and the stripping tendon pulls the tile in reverse to achieve shedding. Matthew et al. [46] connected two sticky tiles with bistable components, investigated the capture of translational and rotating objects by the gripper under the incident conditions of different angles, velocities and angular velocities, and conducted experiments in a microgravity environment to provide an envelope that could be grasped. This shows the advantages of wedge-shaped surfaces for non-cooperative target acquisition in space. As shown in Figure 5a,b, Hawkes et al. [47] designed the mechanism of tendon and double tile, optimized the tile spacing and tendon connection position, and integrated the double-tile mechanism into the quadrotor aircraft to help realize the roosting of the aircraft. And the simulation analysis of three sticky tiles is carried out. The three directions of the adhesive on the three tiles are symmetrical, and the tangential movement of the tiles is controlled to control the adhesion. Modabberifar et al. [48] used shape memory alloy wires to drive the three-tile gripper tangentially, avoiding the risk of tendon loosening over time in tendon control. However, the gripper has seven times the power consumption and a slower response compared to existing grippers. Modabberifar et al. then tried using the Scott Russell mechanism to transfer the load from the center to the three sticky tiles [49], achieving an even distribution of load and increasing the bonding capacity by 19.6–50%. Because the controlled adhesion of the wedge-shaped surface is achieved by shear, less is seen on soft grippers. Hawkes et al. [50] proposed the idea of lateral clamping. As shown in Figure 5c,d, an air bag is mounted on the side arms, and the gravity of the object provides a shearing tendency during gripping. The soft gripper has good adaptability to a variety of shapes, but it is difficult to control.

Climbing robots play an important role in the inspection and maintenance of dams, bridges, hulls, wind turbine blades, space stations and other large structures. The involvement of robots reduces costs and improves efficiency and safety [51]. The application scenario of a climbing robot requires it to have the ability to crawl flexibly on flat or slightly curved surfaces, and its “walk” and “stop” are easy to control. Typically, space non-cooperative target acquisition is where gecko adhesives are thought to shine. The lack of information on the acquisition of non-cooperative targets by rigid robotic arms is easy to cause collision damage, which in turn generates more space debris. Traditional adsorption methods such as electrostatic adsorption and negative pressure adsorption are no longer applicable in the space environment. The adhesion performance of gecko-like dry adhesive in a vacuum is almost unaffected, the gentle capture method is not easy to produce new space debris, and it can be used repeatedly after detachment. In addition, objects in space tend to be in a tumbling state, with angular velocity. The high shear strength of the wedge surface is conducive to racemization [52,53,54]. However, the relevant research still seems to stay in the conceptual design, and there is still a distance from the practical application.

## 3. The Suction Cup Surface

**Figure 6:**
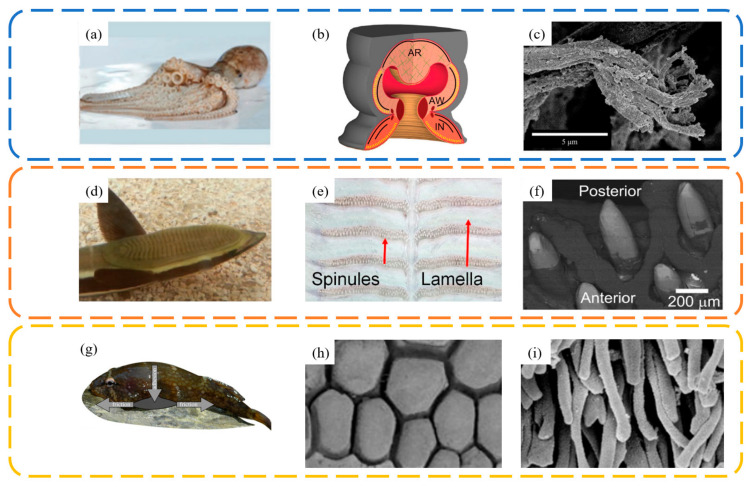
Common bioinspired suction cup prototypes in nature and the fine structures of their functional units. Octopuses use their arms covered with macro suckers to crawl on the rocks in the sea. (**a**) Octopus image. Adapted with permission from Ref. [9]. Copyright 2015, WILEY-VCH Verlag GmbH & Co. (**b**) Sucker image. Adapted with permission from Ref. [56]. Copyright 2013, Tramacere et al. (**c**) Surface microstructure of octopus suckers. Adapted with permission from Ref. [5]. Copyright 2014, Tramacere et al. Remoras attach to sharks using their dorsal adhesive discs consisting of a series of parallel lamellae with spinules. (**d**) Remora image. Adapted with permission from Ref. [57]. Copyright 2014, Material Research Society. (**e**) Spinule distribution image. (**f**) Spinule image. Adapted with permission from Ref. [58]. Copyright 2015, The Company of Biologists Ltd. Clingfish are characterized by ventral adhesive discs covered with papillae for underwater attachment. (**g**) Clingfish image. (**h**,**i**) Papilla image. Adapted with permission from Ref. [59]. Copyright 2013, Wainwright et al.

In the natural world, many aquatic organisms have evolved biological suction cups to adapt to the complex underwater environment. These suction cups enable them to adhere to target surfaces (such as rocks and prey) to enhance their predation abilities and stability in water currents. The surfaces of these biological suction cups often possess micro- and nanostructures that aid in achieving better contact, sealing, and enhanced friction with the target surfaces. Figure 6 summarizes common bioinspired suction cup prototypes: the suction cups on octopus tentacles (Figure 6a–c), the top of a remora’s head (Figure 6d–f), and the clingfish (Figure 6g–i) [55]. These biological suction cups exhibit excellent underwater negative pressure adhesion capabilities. Despite variations in their attachment structures and sizes, micro- and nano-scale protrusions have been found on the edges or inside of their suction cup lips.

For instance, octopuses can adhere to and crawl on rocks, using their tentacles covered in suction cups to capture prey. The hair-like array structures on the edges of their suction cups assist in strengthening adherence to surfaces. Remoras use the suction cup on their head to attach to larger fish like sharks, hitchhiking through the sea. This suction cup contains a series of parallel, spiny lamellae, which, when activated, increase the volume of the sealed chamber to create negative pressure, simultaneously enhancing friction with the attachment surface. Lampreys utilize the suction cup on their ventral throat for adhering to underwater rocks. The hexagonal protrusions tightly arranged in the cup, with tiered villi, increase friction on irregular surfaces. The abalone utilizes its gastropod for both attachment and mobility. Its ability to adhere is remarkably strong, capable of supporting approximately 200 to 300 times its own body weight [60]. The mucus in its adhesion zone is crucial for the successful attachment of the abalone based on capillary forces and viscous forces [61]. These biological suction cups, while differing in morphological structure, all provide sufficient adhesion to contact surfaces. Moreover, the micro/nanostructures on these suction cups contribute to increasing the friction between the cup and the base, preventing slippage at the edges that could lead to detachment. Based on these biological prototypes, scientists have designed and manufactured various suction cups suitable for attachment and grasping on rough surfaces in both underwater and dry environments.

### 3.1. Mechanism

#### 3.1.1. Suction Force

The suction attachment mechanism of various aquatic organisms, despite the differing shapes of their suction cup surfaces, is fundamentally the same. It involves creating a closed attachment surface and maintaining a pressure difference between the inside and the outside. The suction force can be described as:

F=A×ΔP

where *F* is the attachment force, *A* is the area of the suction cup, and ∆*P* is the pressure difference between the outside and inside of the suction cup. The combination of a sealed attachment surface and a pressure differential is what enables these organisms to firmly adhere to surfaces, even under challenging conditions like strong currents or rough surfaces. This mechanism is widely observed across a variety of aquatic species, each adapting the fundamental principle to their unique morphology and ecological needs.

It is noteworthy that the suction force typically only provides a force perpendicular to the contact surface, which is known as the normal force. Its ability to prevent sliding along the contact surface (i.e., movement parallel to the contact surface) is limited. This means that under certain conditions, if the external force is strong enough, the object might slide along the contact surface or detach.

#### 3.1.2. Friction Force

In nature, suction cups can also bear tangential loads. For octopuses, their suction cups are equipped with muscular, flexible walls, which can conform to the shape of the prey or surface they are holding onto. Similarly, remoras use their uniquely evolved suction cups, located on the top of their heads, to attach themselves to larger, moving marine animals. The friction force plays an important role when the suction cup enduring dynamic tangential loads. The friction force can be summarized as:

F=μN

In which, F is the friction force, N is the normal force, and μ is the friction coefficient between the suction cup and the attach surface. Appropriate friction is also the guarantee of suction cup load-bearing. When bearing a load, the frictional force anchors the edges of the suction cup, thereby providing a greater suction force. Biological suction cups achieve enhanced frictional forces through appropriate surface roughness and improved conformity to rough surfaces. The same methods also apply to the design of bionic suction cups.

### 3.2. Manufacturing Process and Surface Capability

Octopuses capture prey through the coordinated action of their suction cups and tentacles, necessitating that the suction cups provide both normal and tangential forces to prevent the prey from slipping. Building on this principle, researchers have developed various octopus-inspired suction cups, attempting to enhance their capabilities through multi-level structures, surface wrinkling, micro-suction cup arrays, and active control mechanisms.

**Figure 7:**
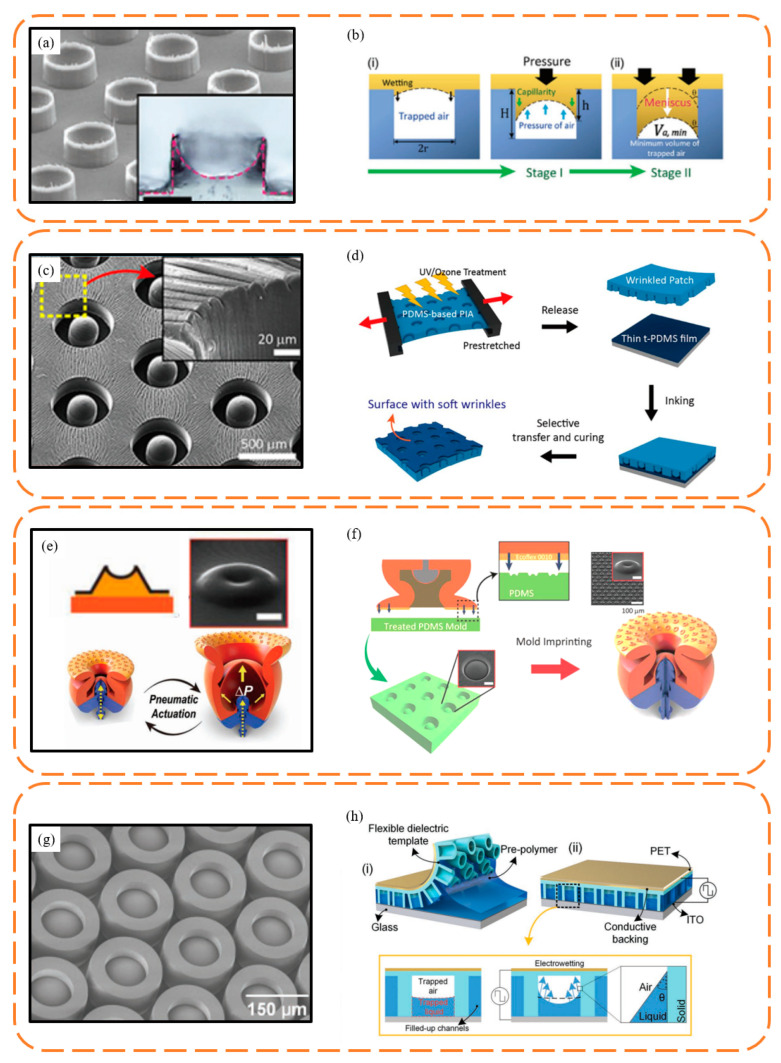
Typical octopus-inspired suction cup surface and the fabrication method. (**a**,**b**) Suction cup surface fabricated by the wetting effect. Reprinted with permission from Ref. [64]. Copyright 2018, Baik et al. (**c**,**d**) Suction cup surface with wrinkles fabricated by exposure to UV/ozone. Reprinted with permission from Ref. [11]. Copyright 2019, American Chemical Society. (**e**,**f**) Suction cup with microsuckers fabricated by double solidification. Reprinted with permission from Ref. [65]. Copyright 2022, Hwang et al. (**g**,**h**) Suction cup surface fabricated by the electrowetting effect. Reprinted with permission from Ref. [66]. Copyright 2022, Wiley-VCH GmbH.

Tramacere et al. [62] employed magnetic resonance images to perform a 3D reconstruction of the octopus suction cup model, which was then used to fabricate centimeter-scale suction cup prototypes. This resulted in achieving a maximum pull-off force of approximately 8 N. Chen et al. [63] used a scalable self-assembly technology to fabricate non-close-packed nanosucker arrays, which could obtain 30 kPa normal force and 12 kPa shear force. They also confirmed that these microdomes prevent the sucker’s outer wall from collapsing, thus stabilizing underwater adhesion. By controlling the meniscus of a liquid precursor in a simple molding process, sucker arrays with 100 µm diameter and 75 µm height were fabricated by Baik et al. [64] (Figure 7a,b). The surface replicated the infundibular and circumferential rim of the octopus sucker, which exhibit highly adaptable and repeatable underwater adhesion. These suckers, with their curved inner cavities, lower the edge modulus for optimal contact with substrates, ensuring a tight seal. It could obtain 30 kPa normal force in a dry condition and 120 kPa in an underwater condition. To achieve a higher shear force underwater condition, a wrinkled octopus suction surface was proposed by Baik et al. [11] (Figure 7c,d). They prestretched the patch and exposed it to UV/ozone to obtain wrinkles and achieved 55 kPa in a dry condition and 40 kPa in an underwater condition. By designing adjustable cavities to increase the pressure difference inside and outside the suction cup, and utilizing secondary molding on the suction cup surface to create smaller-scale suction cups, Hwang et al. [65] enhanced the suction cup’s adaptability to rough surfaces while improving its controllability (Figure 7e,f). This ultimately resulted in achieving a maximum normal force of 29 N in dry conditions and 49 N underwater at an input pressure of 80 kPa. By implementing the electrowetting effect, the microdome curvature ratio of the sucker could be changed and a sucker array with normal force of 86 kPa in air and 61 kPa in water was proposed by Wu et al. [66] (Figure 7g,h).

### 3.3. Applications

**Figure 8:**
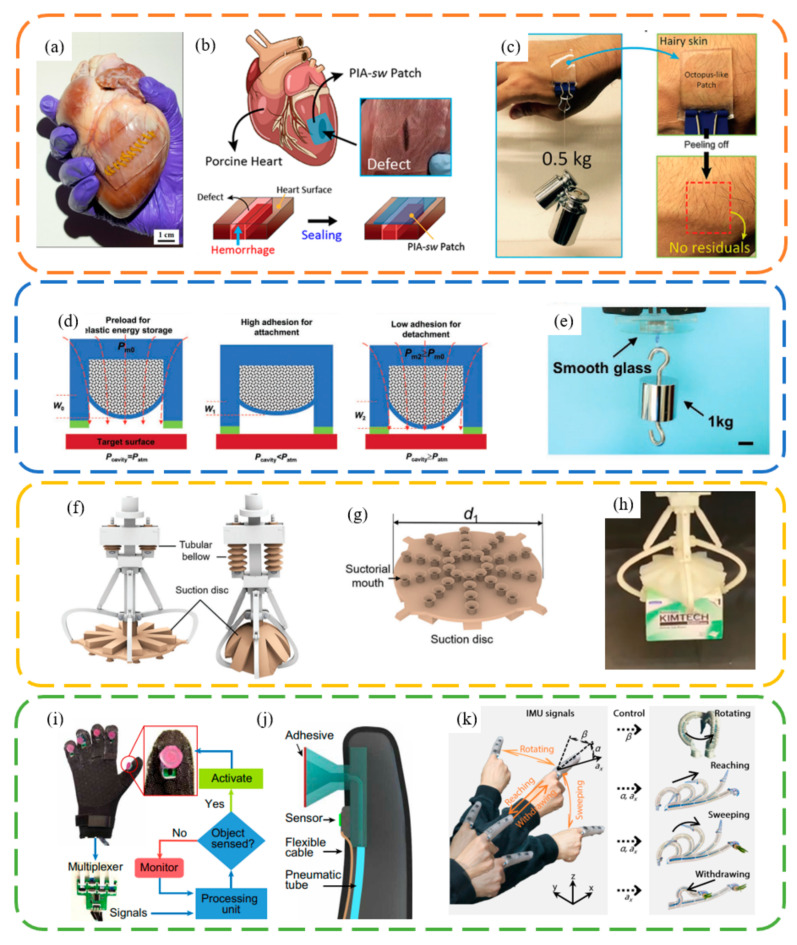
Applications of bioinspired suction cup surface. (**a**–**c**) Heart and skin attachment for medical purposes. Reprinted with permission from [11,63,64]. Copyright 2017, American Chemical Society. Copyright 2018, Baik et al. Copyright 2019, American Chemical Society. (**d**,**e**) Magnetic actuated sucker. Reprinted with permission from [71]. Copyright 2020, Wiley-VCH GmbH. (**f**–**h**) Octopus-inspired gripper. Reprinted with permission from [72]. Copyright 2022, Wu et al. (**i**–**k**) Octopus-inspired adhesives with embedded sensing, processing, and control capabilities. Reprinted with permission from [1,75]. Copyright 2022, Frey et al. Copyright 2023, Xie et al.

Bioinspired suction cups have a wide range of applications, including biomedical uses, smart adhesion methods [67], soft grippers, and intelligent grasping. Chen et al. [63] demonstrated that a nanosucker array can maintain adhesion on a porcine heart, aiding in wound-healing. Baik et al. [11] showcased the use of a microsucker patch in preventing hemorrhage of a porcine heart (Figure 8a). Furthermore, they reported that a patch (2 × 2 cm^2^) could withstand a total weight of 0.5 kg attached to rough skin covered with hairs for 6 h [64] (Figure 8b). These works highlight the potential applications of octopus-inspired microsucker arrays in internal and external wound-healing, electronic skin [68,69], and wearable devices [70] (Figure 8c).

Suction cups, due to their reliance on internal and external pressure differences for load-bearing, typically require valves to regulate the internal pressure for controlled adhesion and detachment in macro-scale applications. This often necessitates the use of pump assemblies and related tubing connections, leading to complex designs and relatively large space occupancy. Therefore, external field control over the adhesion and detachment of suction cups is of significant importance. Wang et al. [71] designed an innovative magnetically actuated adhesive, inspired by the elastic energy storage principle in octopus suckers, as illustrated in Figure 8d,e. Specifically, by attracting the magnet in the upper cavity, an external magnetic field can control the deformation of the elastic film. This alters the volume of the lower empty cavity, allowing the interfacial adhesion force to be modulated by the resulting pressure difference.

Wu et al. [72] showcased a soft gripper inspired by the advanced grasping capability of the glowing sucker octopus (*Stauroteuthis syrtensis*), specifically its unique umbrella-shaped dorsal and ventral membrane between each arm (Figure 8f–h). The unique umbrella-shaped dorsal greatly improved the grasping capability of the suckers. They achieved grasping capabilities for a variety of situations, including multimodal and rough surfaces, flat and scattered objects, as well as moving animals. This soft gripper holds vast potential for applications in underwater fishing and salvage operations, logistics sorting, and the selection and placement of heterogeneous objects on assembly lines.

Combining suction cups with octopus-like arms is an innovative method to enhance grasping capabilities. Mazzolai et al. [73] proposed the design of a conical soft robotic arm equipped with suction cups. This arm is capable of grasping various complex-shaped objects and operating in air, water, and oil, as well as of retrieving items. The suction cups significantly improve the arm’s ability to retrieve objects that would otherwise be impossible to grasp, enhancing the grasping force by up to 1.4 times in air, 2.4 times in water, and 12.5 times in oil. Xie et al. [74] incorporated vacuum-actuated suckers into octopus-inspired conical-shaped actuators. Notably, their findings reveal that due to their increased flexibility, these tapered actuators with suckers exhibit superior gripping power compared to their cylindrical counterparts and require significantly greater forces for detachment from both flat and curved surfaces. They demonstrated that by selecting suitable taper angles, these tapered actuators with suckers can grip, move, and position a remarkably wide range of objects with flat, nonplanar, smooth, or rough surfaces, as well as retrieve objects through narrow openings.

Frey et al. [75] have synergized switchable, octopus-inspired adhesives with embedded sensing, processing, and control capabilities for robust underwater manipulation (Figure 8i,j). The adhesion strength of these adhesives can be altered over 450 times from the ON to OFF state in less than 50 milliseconds, across many cycles, using an actively controlled membrane. Their bioinspired “nervous system” autonomously detects objects and triggers the switchable adhesives. Xie et al. [1] developed an electronics-integrated soft octopus arm (E-SOAM) adept at reaching, sensing, grasping, and interacting within a substantial operational domain (Figure 8k). By merging a sensor-equipped distal section with a soft arm, the E-SOAM is capable of executing a sequence of reaching, grasping, and withdrawing motions, extending up to 1.5 times its original arm length, mirroring the capabilities of its biological counterpart. This technology is further enhanced with a wearable finger glove that replicates suction sensations, enabling a human operator to remotely and interactively control the robot’s movements, both in and out of plane, and its grasping actions, in both aerial and underwater environments, using just a single finger.

## 4. Claw Thorn Surface

**Figure 9:**
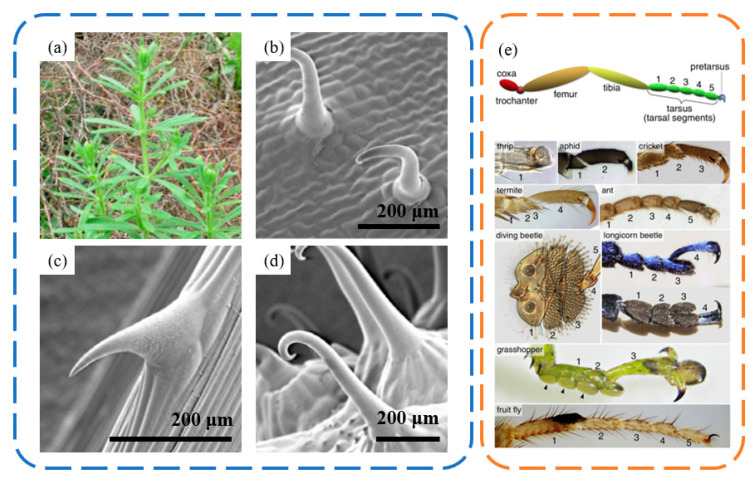
The structures of Galium aparine spines and insect foot claw spines. (**a**) Photo of Galium aparine. (**b**–**d**) SEM micrographs of the adaxial leaf surface, stem surface and mericarp surface. Reprinted with permission from [79]. Copyright 2014, Taylor Francis Group In Journal of Adhesion Science and Technology. (**e**) The structures and attachment levels of claw spines on the feet of various insects. Reprinted with permission from [78]. Copyright 2016, Elsevier Inc.

Claw-like attachments are commonly found in nature, where organisms use these structures to penetrate or mechanically interlock with surfaces. In plants, such structures are often seen in certain seeds, typically flexible, enabling them to adhere to each other or to fur [76,77]. Gorb et al. [76,77] investigated the adhesive properties of four plant species with claw-like structures (*Agrimonia eupatoria*, *Circaea lutetiana*, *Galium aparine*, and *Geum urbanum*). Figure 9a–d display the microscopic structures of these plants, covered with hook-like flexible claws, each having a terminal size on the scale of 10 μm. The detachment force between a single claw and an iron wire loop was measured using an experimental setup. The detachment primarily occurs due to the elastic deformation of the claw. Therefore, this mode of attachment, relying on mechanical interlocking through flexible claw shapes, is significantly influenced by the structural size and mechanical properties of the claws. Attachment mainly relies on the mechanical engagement between the micro-claws and rough peaks, while detachment requires forceful pulling. Hence, replicating this micro-claw attachment necessitates the use of materials with high toughness, as these claws are prone to breakage and gradual loss of adhesive ability.

Claw-like attachments are even more common in animals, particularly in insects, where many have claw structures at the ends of their limbs [78] (Figure 9e). These typically hook-shaped claws interact mechanically with the rough peaks on surfaces or can directly penetrate into softer contact surfaces. This enables insects to stay or even walk on vertical or ceiling surfaces with rough textures. When adhering to vertical walls, insects rely on unidirectional claws for the necessary attachment strength. However, for ceiling surfaces, they require normal adhesive forces. Unlike geckos, which adhere to ceiling surfaces through van der Waals forces, insects predominantly use claw gripping for attachment to rough ceiling surfaces.

**Figure 10:**
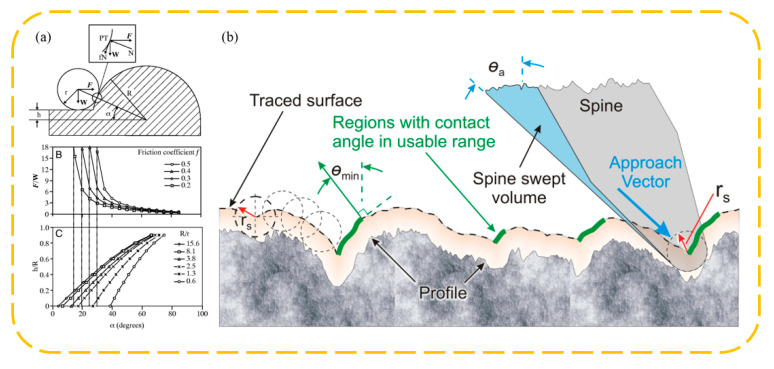
Claw spine attachment mechanism and theoretical model. (**a**) The claw–spur ball/ball contact attachment model proposed by Dai et al. Reprinted with permission from [6]. Copyright 2002, Company of Biologists Ltd. (**b**) The ball/rough wall attachment model proposed by Asbeck et al. Reprinted with permission from [86,87]. Copyright 2010, Asbeck et al. Copyright 2006, SAGE Publications.

Research has shown that some beetles in the Scarabaeidae family have robust reversed spine structures at the ends of their legs or tarsi [80], which work in tandem with their terminal claws to enhance their grip, particularly on ceiling surfaces. Song et al. [81] also discovered that insect claws can synergize with their adhesive pads, increasing their adherence capabilities on various rough surfaces. As shown in Figure 10b, comparing the morphology of the adhered surfaces with the size of the insect claws reveals a mutual correspondence. At very low surface roughness, adhesion predominates, while at higher roughness, mechanical interlocking by claws provides the primary adhesive force. Some aquatic organisms also use mechanical interlocking to adhere to surfaces underwater, such as rocks. As early as 1924, researchers discovered that mayfly nymphs could use their foot claws to stay on surfaces even under strong water currents [82,83,84]. Additionally, Johal et al. [85] suggested that the claw-like structures on the abdomen of Glyptothorax Garhwali Tilak assist in their mechanical interlocking attachment to the ground. Such research progress underlines the critical role of claw-like structures in the diverse adhesion strategies of both terrestrial and aquatic organisms, showcasing nature’s ingenuity in adapting to a variety of environmental challenges.

### 4.1. Mechanism

The theory of mechanical interlocking attachment through biological claw-like structures is primarily based on rigid body mechanics, taking into account the forces on claw tips with certain shapes on rough surfaces. Dai et al. [6] measured the tangential adhesion properties of claw tips from Scarabaeidae beetle legs on various sandpaper surfaces and proposed a mechanical model of claw–surface contact. They analyzed the relationship between the structural dimensions of the claws, the size of the rough peaks, and the coefficient of friction with maximum tangential adhesion force. As shown in Figure 10a, this contact model simplifies the interaction between the claw tip and the rough peak on the sandpaper surface to a sphere–sphere contact. Here, the radius of the claw tip is r, the radius of the rough peak is R, the distance of the spherical cap center from the surface is h, the angle between the line connecting the contact points and the horizontal is α, the forces acting in the horizontal and vertical directions are *F* and *W*, respectively, and the coefficient of friction between them is μ. Taking into account the sliding of the tip on the surface, the force balance condition yields the following equation:

$$\frac{F}{W}=\frac{cos\alpha +\mu sin\alpha }{sin\alpha -\mu cos\alpha }=\frac{1+\mu tan\alpha }{tan\alpha -\mu }$$

where α and h satisfy:

$$sin\alpha =\frac{r+h}{r+R}=\frac{\frac{r}{R}+\frac{h}{R}}{\frac{r}{R}+1}$$

From this, we can establish the relationship between the load ratio *F*/*W*, the spherical cap offset ratio $\frac{h}{R}$, and the angle α, leading to the results presented in Figure 10a. It is observed that the coefficient of friction greatly influences the tangential adhesion of the claw. A higher coefficient of friction means a larger α can be achieved during self-locking (where the load ratio *F*/*W* is infinitely large), allowing mechanical interlocking even with larger claw tip radii. If the friction coefficient is relatively low, specifically μ<tanα, frictional self-locking cannot occur. In such cases, to achieve tangential adhesion, a normal force must be applied; otherwise, detachment will occur. Without frictional self-locking, only a normal force can provide tangential adhesion, which rapidly decreases as the angle α increases. For climbing vertical walls and ceilings, a normal adhesive force from the wall is often required. In the absence of frictional self-locking, this adhesive force cannot be provided.

Building on this, Asbeck et al. [86,87] considered the adhesion of claws on general rough surfaces (as shown in Figure 10b), discussing the matter using the surface contour normal angle *θ* instead of α. They defined the load ratio *W*/*F* as tan *θ*load. From this, the minimum normal angle *θ*min required for mechanical interlocking can be determined as:

$$\theta_{min}=\theta_{load}+arccot\mu$$

This formula serves as a criterion for determining the potential adhesion sites on rough surfaces.

### 4.2. Applications

**Figure 11:**
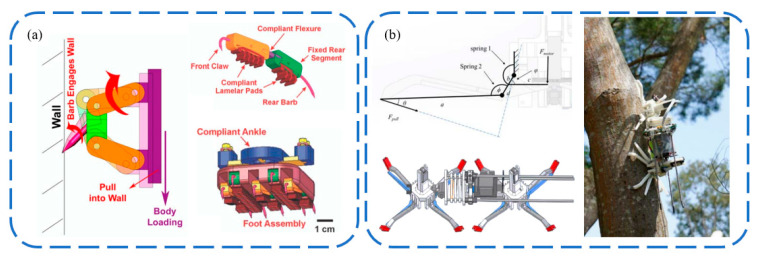
Penetrating bionic claw spine attachment. (**a**) Penetrating claw for soft wall surfaces. Reprinted with permission from [8]. Copyright 2005, Springer-Verlag Berlin Heidelberg; (**b**) Tree-climbing robot with penetrating bionic claw. Reprinted with permission from [88]. Copyright 2014, IEEE.

Research on biomimetic claw attachments based on penetration methods is limited. This approach is suitable for surfaces that can be penetrated, such as rubber or tree bark. As illustrated in Figure 11a, William et al. [8] were the first to propose a type of biomimetic claw attachment for soft surfaces, exploring how the shape of the needles affects the maximum penetration force and detachment force. Such attachment claws require a significant preload to ensure adequate penetration depth and adhesion. Moreover, the tangential adhesion force is much greater than the normal adhesion force, making them primarily suitable for tangential attachment. Subsequently, Liu et al. [88] developed a dual-footed tree-climbing robot, employing retractable passive penetration claws (Figure 11b). However, this design resulted in relatively low penetration depth and adhesion force. Reports on biomimetic claw attachments for penetrating soft surfaces like rubber are still lacking, indicating a need for the development of new attachment claws to achieve adhesion on such surfaces.

Biomimetic claw attachment methods based on mechanical interlocking are well-suited for rough surfaces, such as those with marine biofouling. Addressing applications like climbing rough walls, grasping objects on rough surfaces, and asteroid rock sampling, numerous biomimetic claw attachment devices and wall-climbing robots have been developed domestically and internationally.

**Figure 12:**
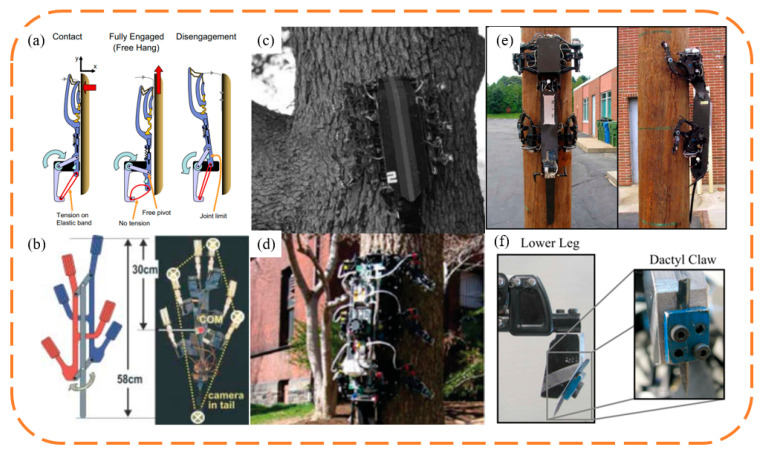
One-way bionic claw spine attachment. (**a**,**b**) SpinyBotII vertical wall-crawling robot. Reprinted with permission from [90]. Copyright 2005, IEEE; (**c**) RiSE V1. Reprinted with permission from [91]. Copyright 2005, Society of Photo-Optical Instrumentation Engineers; (**d**) RiSE V2. Reprinted with permission from [92]. Copyright 2008, Wiley Periodicals, Inc; (**e**,**f**) RiSE V3. Reprinted with permission from [93]. Copyright 2009, IEEE.

The rise of biomimetic claw attachment began in 2005 with the RiSE (Robots in Scansorial Environments) project, which aimed to develop robots capable of climbing on general surfaces. Figure 12 showcases some of the project’s typical outcomes, as well as other unidirectional biomimetic claw attachment devices inspired by the project. Asbeck et al. [87,89,90] developed the first vertical wall-climbing biomimetic claw robot, SpinyBotII (Figure 12a), which climbs with a fixed triangular gait using six claw attachment devices, each comprising ten independently movable claws, created using Shape Deposition Manufacturing (SDM), which blends flexible and hard materials. Concurrently, Autumn and Saunders from Boston Dynamics collaborated on three generations of RiSE wall-climbing robots (Figure 12c–f). RiSE V1 [91] in Figure 12c, also made using SDM, did not consider a flexible adaptive design for the claws. The robot had more degrees of freedom than SpinyBotII, with a two-degree freedom in each leg, enabling it to climb and adhere to curved surfaces like trees. Spenko et al. [92] then developed RiSE V2, based on RiSE V1, with flexible structures similar to SpinyBotII, achieving multi-level passive adaptation synergizing flexible claws with underactuated spring-damping leg mechanisms. The robot used leg force control and gait planning for sensing and control, enabling RiSE V2 to achieve reliable long-distance climbing on walls. Haynes et al. [93] further developed RiSE V3, a quadruped robot capable of dynamic, high-speed climbing on tree trunks. RiSE V3 utilized a new linkage leg mechanism and high-energy-density power transmission, allowing the robot to climb at speeds of up to 21 cm/s, and it used robust claws for strong adhesion to tree trunks. Following this, many researchers adopted biomimetic claw attachment methods to develop wall-climbing robots. To achieve faster vertical wall-climbing, Daltorio et al. [94,95] developed several wall-climbing robots with a wheel–leg hybrid configuration, and Clark et al. [96] mimicked the locomotion of insects and geckos to develop a similar hybrid robot capable of rapid vertical climbing.

**Figure 13:**
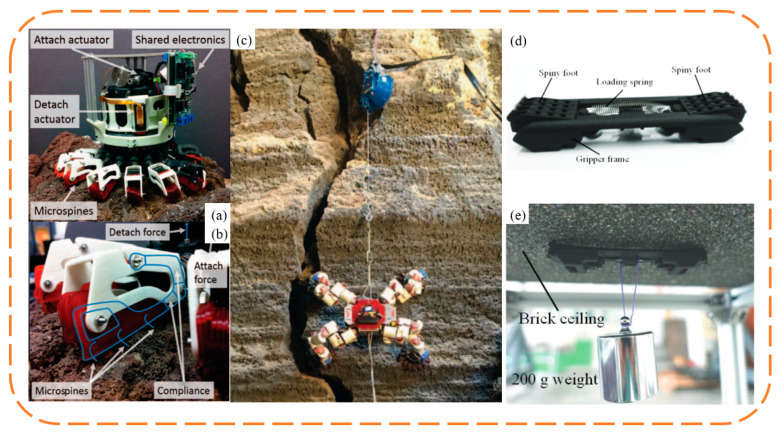
Bionic claw spines robot with counter claws. (**a**–**c**) LEMUR 3 robot. Reprinted with permission from [3]. Copyright 2017, IEEE; (**d**,**e**) Crawler-type wall-climbing robot with counter-grabbing claws. Reprinted with permission from [103]. Copyright 2020, Jilin University.

For ceiling rough surface adhesion, researchers, inspired by insect foot claw gripping, developed various gripping biomimetic claw attachment devices for wall-climbing robots, as shown in Figure 13. Parness et al. [97] developed several gripping biomimetic claw attachment devices. To meet the needs of asteroid sampling, Parness et al., based on the flexible claw mechanism from the RiSE series, constructed a ring array of gripping attachment claws, providing adhesion for rock drilling under microgravity conditions [98], and they applied these claws to the wall-climbing robot LEMUR IIB [99], enabling it to climb on ceiling surfaces with the aid of pulling forces. Subsequent iterations and improvements [100,101] included using parallelogram and annular flexible structures made of aerospace materials instead of the SDM multi-material flexible structure. These improved attachment claws were applied to LEMUR 3 [3], enabling it to demonstrate climbing on cliff faces (Figure 13c). LEMUR 3′s legs consist of modular 7-degree-of-freedom mechanical arms, with single-axis force sensors at the ends to measure the forces during climbing, enabling real-time control of the robot’s legs for reliable climbing. Liu Yanwei et al. [102] developed a bipedal wall-climbing robot that is capable of transitioning from vertical walls to ceiling surfaces, mimicking the motion of inchworms with three degrees of freedom. The robot’s attachment claws, inspired by the foot structure of the Oriental beetle, were manufactured using Selective Laser Sintering (SLS) to create a chevron-shaped flexible claw structure. Additionally, this flexible claw structure design was applied to a tracked ceiling-walking robot [103], with symmetrically arranged claw units on the tracks attaching to and detaching from surfaces via tracks on the frame, allowing the robot to crawl smoothly on ceiling surfaces, although manual intervention is needed to open the claw units for initial attachment.

**Figure 14:**
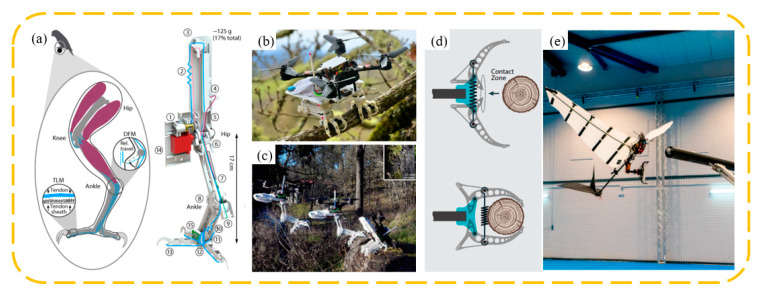
The schematic structure (**a**) of a stereotyped nature-inspired aerial grasper and landing images (**b**,**c**). Reprinted with permission from [104]. Copyright 2021, Roderick et al. (**d**) Bistable claw configuration, before and after contact. (**e**) Robot in the approach phase to the branch. Reprinted with permission from [105]. Copyright 2022, Zufferey et al.

Bird-inspired dynamic grasping and perching claws offer unique advantages, as they can capture objects moving at certain speeds and achieve rapid and effective grasping in the short duration of contact between the claw and the object. Roderick et al. [104] developed a biomimetic robot capable of dynamically perching on complex surfaces and grasping irregular objects (Figure 14a). To handle high-speed impacts, the robot’s legs passively convert the impact energy into a grasping force. Simultaneously, its underactuated grasping mechanism can envelop irregularly shaped objects in less than 50 milliseconds. Zufferey et al. [105] engineered a flapping-wing robot capable of grasping a branch within 25 milliseconds and subsequently reopening (Figure 14b). They also tested this approach with a 700 g robot, successfully demonstrating the first autonomous perching flight of a flapping-wing robot on a branch.

## 5. Composite Biomimetic Adhesive Surface

**Figure 15:**
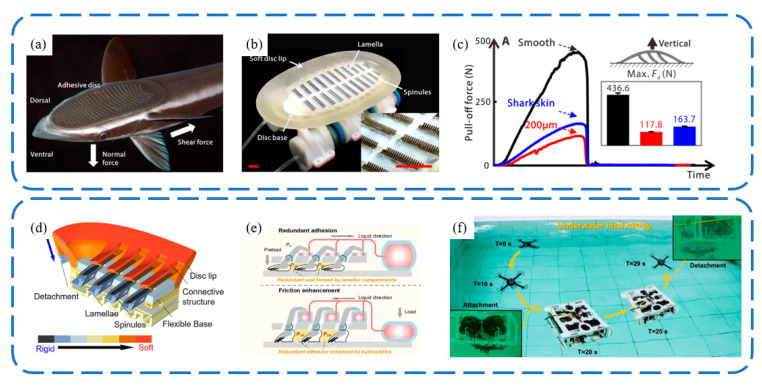
Biorobotic adhesive discs inspired by the remora suckerfish. (**a**) Photo of remora suckerfish. (**b**) Photo of biorobotic adhesive disc. (**c**) Pull-off force of biorobotic adhesive disc. Reprinted with permission from [106]. Copyright 2017, Wang et al. Schematic structure of the biorobotic adhesive disc (**d**,**e**) and underwater hitchhiking demonstration (**f**). Reprinted with permission from [109]. Copyright 2022, Li et al.

Wang et al. [106] introduced the design of a biologically inspired, multimaterial biomimetic remora disc, based on detailed morphological and kinematic studies of the slender sharksucker (*Echeneis naucrates*) (Figure 15a). They employed multimaterial three-dimensional printing techniques to create the main disc structure, featuring a stiffness range spanning three orders of magnitude. To replicate the functionality of the remora’s lamellae, carbon fiber spinules with a base diameter of 270 μm were crafted using laser-machining techniques and affixed to lamellae controlled by soft actuators. This biomimetic prototype can adhere to various surfaces and generate a considerable pull-off force of up to 436 N. The rigid spinules and the soft material on the lamellae engage with surfaces upon rotation, emulating the action of live remora discs. The biomimetic kinematics lead to significantly increased frictional forces across the disc on substrates with varying roughness. Lee et al. [107] created a biomimetic remora suction cup using PDMS casting, achieving an underwater normal adhesion strength of 266.8 kPa and a tangential strength of 194.2 kPa. The flexible micro-spine structure within the suction cup can increase the contact area with the base during tangential deformation, thereby enhancing its tangential adhesion capability. This mechanism is similar to the adhesion principle of biomimetic gecko-inspired wedge array surfaces [32,108]. Following these advancements, Li et al. [109] investigated the adhesion capabilities of the biomimetic remora disc in air (Figure 15b). Integrated with a rotor-based aerial–aquatic robot, it could swiftly attach to and detach from challenging surfaces, including curved, rough, incomplete, and biofouled surfaces, both in air and underwater. Additionally, the system achieves prolonged adhesion with minimal oscillation.

As depicted in Table 1, the basic bioinspired surfaces (wedge-shaped surface, suction cup surface, and claw thorn surface) exhibit suitable roughness and curvature for specific target surfaces, as constrained by the characteristic length of the surface features. Simultaneously, their primary bearing directions and suitable operating environments vary. The Remora-inspired adhesive disc combines the features of the sucker surface (disc) and thorn surface (spinules), thereby enhancing the maximum tangential force on the disc. This insight inspires the idea that when a basic bioinspired surface falls short of meeting the load requirements for a gripping surface, a composite biomimetic adhesive surface can be designed by combining various basic bioinspired surfaces. Consequently, the composite biomimetic adhesive surface may showcase superior adaptability to the surface roughness and curvature radius, along with the capability for multidirectional bearing—attributes that might not be inherently present in the basic bioinspired surface.

**Table 1:** Bioinspired surfaces and their main loading direction, usage environment and estimation of the target surface curvature and roughness based on its feature length.

Ref.	Bioinspired	Shape	Feature Length *a* (μm)	Target Curvature	Target Surface Roughness	Main Loading Direction	Environment
[1,11,63,64,65,66,69,72,73,74,75,110]	Octopus	Sucker	Sucker radius: 0.1~10^5^	>*a*	<<*a*	Normal	Underwater/Air
[20,26,32,41,43,49,111,112,113,114]	Gecko	Wedge shaped	Height: 10^1^~10^3^	>*a*	<<*a*	Tangential	Air/Vacuum
[6,80,86,87,115]	Beetle	Thorn	Tip radius: 1~10^3^	>*a*	>*a*	Normal/Tangential	Underwater/ Air/Vacuum
[104,105]	Bird	Claw	Claw length: ≈10^4^	≈*a*	<*a*	Normal/Tangential	Underwater/ Air/Vacuum
[106,109]	Remora	Sucker + Thorn	Sucker radius: *a* ≈ 10^5^ Tip radius: 1 < *b* < 10^3^	>*a*	<<*a* >*b*	Normal/Tangential	Underwater/Air

## 6. Conclusions and Perspective

The astonishing switchable adhesion abilities in biology, based on interactions (e.g., van der Waals force, capillary force, suction force, interlock force, etc.) generated at the soft adhesion contacts between micro/nano structures and substrates, provide abundant inspiration for engineering artificial surfaces with superior functions in an elegant paradigm. Herein, we review recent advances in biological switchable adhesive surfaces (geckos, octopuses, remoras, beetles, eagles) and the corresponding artificial adhesives from the perspective of the surface type. We discuss the adhesion mechanisms of biological adhesive surfaces based on the different types. Additionally, the design principles and basic preparation methods of these biomimetic adhesive surfaces are summarized. Pioneering studies have demonstrated the extraordinary adhesive performances of bioinspired adhesives in gripping tasks, climbing robots (e.g., switchable adhesion and strong interfacial adhesion), and externally controlled adhesive surfaces (achieving strong adhesion and easy detachment through external field control). Drawing inspiration from remoras’ adhesive discs, a novel composite biomimetic adhesive surface is presented to elevate the adhesion capabilities. Integrating features from fundamental bioinspired surfaces—wedge-shaped, suction cup, and claw thorn—the composite biomimetic adhesive surface is poised to exhibit enhanced adaptability to surface roughness and curvature radius. Additionally, it may manifest the ability for multi-directional bearing, potentially surpassing attributes found in the basic bioinspired surface. For gripping tasks, bioinspired surfaces and their gripping systems have some challenges:

a. Composite Biomimetic Adhesive Surface:

The composite biomimetic adhesive surface holds the promise of overcoming the technical limitations imposed by fabricating enhanced single-type biomimetic adhesive surfaces under existing conditions. By integrating the advantages of various biomimetic adhesive surfaces, it could achieve greater adaptability to rough/curved surfaces and diverse working environments and improve its multidirectional bearing capacity. However, the manufacturing method presents a significant challenge for creating a composite biomimetic adhesive surface, which often demands more sophisticated manufacturing techniques. The design and fabrication of microscale composite biomimetic adhesive surfaces still require ongoing exploration.

b. Rapid and Robust Gripper:

In nature, birds can quickly grab moving prey, beetles can move rapidly on rough surfaces, and geckos can run on inverted walls. Natural adhesion and detachment behaviors are rapid and robust. In the existing gripper designs, improving the contact quality between bioinspired surfaces and target surfaces to achieve high load-bearing often means poorer contact quality and lower robustness in rapid contact and gripping. Therefore, a gripper that is both fast and robust will better leverage the load-bearing capacity of bioinspired surfaces, representing an important direction for future research.

c. Active Perception for Dexterous Grasping:

In daily life, grasping is often closely associated with perception. By perceiving the pose or morphological features of the target object, better completion of grasping tasks can be achieved. Based on perception and control, combined with the performance of bioinspired adhesive surfaces, a thorough understanding of the adhesion behavior patterns of bioinspired adhesive surfaces can provide an effective approach to achieve dexterous grasping tasks.

## Data Availability

Not applicable.
